# An integrated psychological strategy for advanced colorectal cancer patients

**DOI:** 10.1186/1477-7525-4-9

**Published:** 2006-02-06

**Authors:** Patrizia Pugliese, Maria Perrone, Enrica Nisi, Carlo Garufi, Diana Giannarelli, Andrew Bottomley, Edmondo Terzoli

**Affiliations:** 1 Service of Psychology, Regina Elena Cancer Institute, Via Elio Chianesi, 53, 00144 Rome, Italy; 2 S.C. Medical Oncology "C", Regina Elena Cancer Institute, Rome, Italy; 3 Biostatistical Unit, Regina Elena Cancer Institute, Rome, Italy; 4 European Organisation for Research and Treatment of Cancer, EORTC Data Center, Brussels, Belgium

## Abstract

**Background:**

There is evidence regarding the usefulness of psychosocial intervention to improve health related quality of life (HRQOL) in adult cancer patients. The aim of this report is to describe an integrated approach and to evaluate its feasibility in routine clinical practice in 98 advanced colorectal cancer (ACC) patients during chronomodulated chemotherapy.

**Methods:**

A prospective non-randomised design was developed and applied in a cancer out-patient setting. The intervention consisted of an integrated approach, whereby the psycho-oncologist had an active role in the health care team with the physician and routinely included psychological understanding in the medical treatment program. The psychological evaluation assessed: a) adaptation, awareness, psychopathological disorders through a psychodynamic interview; b) anxiety and depression using the HAD scale; c) subjective perception of care quality through a structured interview and d) HRQOL evaluation assessment with the EORTC QLQ C30. Outcomes data were collected before and after 18 weeks of chemotherapy.

**Results:**

After 18 weeks of chemotherapy a significant improvement of adaptation and awareness was observed. The HADs results showed a significant decrease in anxiety when compared to pre-treatment. The structured interview showed a significant increase of patients who positively experienced the impact of medical treatment on HRQOL, anxiety, depression, interpersonal relationships, free-time and who positively experienced the care quality. Indeed, a majority of patients positively experienced the team relationship modality during the whole treatment. All scales on the EORTC questionnaire remained unchanged during the entire treatment.

**Conclusion:**

Our results suggest that it is feasible to carry out an integrated approach during chemotherapy. These results seem to support the integrated approach as a tool in aiding advanced colorectal cancer patients' ability to cope with their diagnosis and treatment although an appropriately designed study is required to confirm this.

## Background

The cancer experience consists of predictable events which, generally, are described on the disease continuum [1]. These events begin with the diagnosis, followed by treatment, remission, recurrence or progression, and then the terminal phase. Cancer and its treatment, whether it is surgery, chemotherapy, or radiation therapy, is perceived as a crisis which consists of both the difficulty of integrating cancer diagnosis into a patient's life and the necessary adjustments to the different phases of the disease and treatment.

Different psychological and psychotherapeutic interventions aim at improving quality of life, reducing psychological morbidity and facilitating crisis adaptation. Two main approaches are reported in the literature: informative educational programs [2,3] and psychotherapeutic interventions. Psychotherapeutic interventions are carried out on either an individual or group basis and use cognitive-behavioural [4-8] and psychodynamic models [9-15]. Controlled experimental trials have been more frequently carried out with the former. Most of these studies showed a significant improvement in psychiatric symptoms or social adjustment as reported by Greer and Moorey [16-18].

From a psychodynamic perspective, the patient actual functioning in terms of past experiences and interpersonal relationships was examined [19-21]. Roth and Fonagy [22] stressed that no controlled trial with psychodynamic therapy had been performed and there is limited data available regarding its efficacy. In the cancer setting, cognitive or dynamic psychotherapy have a very flexible approach with the focus on medical illness and QoL issues. Despite these characteristics some problems remain. Patients may find it difficult to attend a regularly structured intervention or to accept a traditional psychological intervention because these can be both emotionally and physically draining. These problems are more evident when the intervention may need to consider short-term life expectancy, along with HRQOL, as in the case of advanced cancer patients.

In clinical practice, physicians treat the disease, prescribe chemotherapy and refer patients with severe psychological symptoms to psychiatric/psychology professionals whenever available. Moreover, in this context the majority of patients do not receive any psychological support and their psychological needs are not taken into account [23]. An integrated approach, where the psycho-oncologist takes an active role in the health care team with the physician and integrates psychological understanding, with a dynamic background directly into routine care from the start, should offer advanced patients more opportunities of support.

The dynamic background provides a point of view for clarifying the onset of psychological symptoms in response to cancer crisis, the meaning of compliance and non compliance with treatment and a perspective on the doctor-patient relationship that is useful for understanding and resolving conflict.

The integrated approach was utilised in advanced colorectal cancer (ACC) patients who were undergoing chemotherapy on an out-patient basis. All of them received a four-month treatment of chronomodulated chemotherapy with computer-programmable external pumps [24]. In many patients, the presence of a portable pump internally leads to an ambivalent object relationship. The pump can be considered a good object when it provides survival and well-being, a demanding object when it requires continual dependence and a bad object when it results in side effects and a threat for psychophysical well-being. Furthermore, the presence of a porth-a-cath can cause phenomena of psychological non compliance, frequent in patients with an excessively rigid, fragmented or non clearly defined body image. This medical treatment and the complexity of psychological response to it, could determine high levels of distress and a non compliance to care with a decrease of survival and a worsening of HRQOL. The objective of this paper was to describe this integrated approach and to evaluate its feasibility in routine clinical practice in ACC patients submitted to chronomodulated chemotherapy.

## Methods

This is a feasibility study, a prospective, non randomised design with no control, and it was used to test the delivery of the integrated approach.

Eligible patients were ACC patients suitable for chemotherapy with a life expectancy of at least six months as assessed by the treating oncologists. All patients received a 5-fluorouracil and folinic acid based regimen ± oxaliplatin [25,26]. All patients were treated with a chronomodulated infusion of the three drugs by means of computer-programmable portable external pumps. Each course lasted one week and was repeated every three weeks. This treatment offers patients a 40–50% response rate, lasting 6–9 months with a median survival of 18–20 months. Main toxicity are oral mucositis and diarrhoea, affecting 30% of patients, while hematological toxicity is very rare. The psychological intervention was considered a standard part of patient care. Verbal informed consent to the medical therapy and to the psychological intervention was obtained from all patients. All the patients were recruited at the Regina Elena Cancer Institute, Rome where they received treatment.

### The integrated approach

The integrated approach provided consisted of the following characteristics:

#### a) Primary care

The intervention was directed at all patients who were confronted with a crisis related to the diagnosis of an advanced disease and to a chemotherapy treatment with the aim of anticipating difficulties and intervening preventively.

Such a crisis could determine behaviour characterised by anxiety, depression, aggressiveness and "helplessness and despair", which often leads to the utilisation of non adapted defences [27]. This condition requires us to take care of all physical and psychological needs to prevent psychological morbidity from the first consultation.

#### b) Integrated

From the start of medical treatment, the integration of 2 health workers, a medical oncologist and a clinical psychologist, who become the patient's main reference point throughout, guarantees two conditions:

1) the structuring of an initial setting at first consultation (basic triangular situation) where the patients can be accepted, listened to and understood and where they can express themselves, recognise their psychophysical needs, and find a first, possible, response to them.

2) The building of a therapeutic relationship, for an adequate communication modality, throughout the course of medical treatment

The presence of 2 health workers from the beginning permits patients to use a dependent relationship modality delegating the solution of their psychophysical needs to these health workers. The acceptance of dependence and consecutive regression could help to decrease anxiety and the utilisation of primitive defences could restore self-mastery.

The integrated approach was developed in different communication phases:

Physicians and psychologists are together in the same office at the first medical consultation when the therapeutic strategy is proposed. The oncologist introduces the psychologist to the patient as a co-therapist in the medical treatment to respond to physical and psychological needs.

The oncologist asks the patient for both medical and psychological treatment consent and informs the patient that the psychological intervention includes clinical interviews and psychometric tests. The presence of the two health workers allows observation of patient first impact with the communication of the diagnosis of advanced disease and of treatment. This combined approach is repeated during all the courses of chemotherapy.

Outside the medical room, the psychologist continues to observe the patient's relationships with both the family and the nurses.

A psychological evaluation, carried out in the psychologist's office, consisting of a descriptive diagnosis according to DSM III-R [28] criteria and of a psychodynamic diagnosis aims at integrating the existing medical condition within the previous patient personal history. The psychodynamic diagnosis helps to understand the meaning of the disease and its treatment, and to design a supportive-expressive intervention modulated on psychological and medical status.

#### c) The psychological supportive-expressive intervention

was intended to favour the expression of emotions regarding cancer and its wide ranging effects on patients' lives (physical, emotional, social and spiritual), about losses related to disease, about medical treatment (expectations, side effects, tumor response) and, also, about the difficulties with health workers and relatives.

A brief focused intervention was offered to patients at high risk for severe psychological distress [29,9-13].

The psychological intervention was carried out by two Ph.D. specialists trained in clinical psychology with at least five years clinical experience and a dynamic training upon which patient understanding is based. The two psychologists alternated in the specific functions of support and research (test administration). The psychologist who was involved in test administration was not involved in the supportive function to the same patient and vice versa. The presence of two psychologists represented a quality control procedure by means of reciprocal supervision.

#### d) Support for the health care professionals

The integrated approach also represented a reciprocal support for the involved health care professionals. This is important when in a progressive disease, in the absence of response to anticancer therapy, the decision to stop active treatment is needed. Reciprocal support is based on daily and weekly meetings/discussions between the oncologists and psychologists organised to discuss staff and clinical problems (progressive disease, changes of medical treatment, patient negative perception of the relationship with health professionals). Daily work in the out-patient setting aimed at the construction of a significant interpersonal relationship with the patient. In the weekly meeting, the observations of the 2 psychologists were constantly reported and shared by oncologists and psychologists, caring for the patients.

This confrontation allows for a continual understanding of the patient's clinical and psychological situation so assuring effective psychological and/or medical strategies.

When structuring the physician-patient relationship, the oncologist must be able to provide the patient with emotional support so that a relationship of trust may be established to facilitate compliance to medical treatment. Within this relationship, the way the oncologist communicates with the patient is of the utmost importance. Information must be clear, respectful of the patient cultural level and defence mechanisms, detailed for instrumental devices, probability of response to treatment and side effects. This should guarantee a personalised relationship that is able to respond to the patient's inner needs.

### Outcome measures

Outcomes measures were collected at baseline and after 18 weeks of chemotherapy.

#### Descriptive diagnosis

according to DSM III-R criteria;

#### Adaptation and awareness

This evaluation was conducted in the psychologist's office before beginning medical therapy. It consisted of a semi-structured psychodynamic interview with two or more meetings with the patient. Adaptation was defined as the redefinition of patient own personal identity threatened by the disease. The patients who used defence mechanisms such as repression, negation and projection were considered adapted patients. The patients who used splitting, denial and projective identification were considered non-adapted patients. The awareness of the type and stage of the disease was interpreted as the capacity the patients have to confront themselves with the image of their health state. The patients are on a continuum with regard to their conscious knowledge of their illness. This continuum ranged from those who appeared to be quite unaware of being seriously ill, to those who clearly knew their illness and all of its implications. Between these two opposed limits there were intermediate levels of knowledge, which varied from mere suspicion of cancer to clear-cut intellectual awareness of illness, though without full emotional understanding. We classified patients into two different levels of awareness: aware patients and unaware patients. The latter included the patients with an absence of conscious awareness and not those with an intermediate awareness. Adaptation was not always related to awareness. The connection between the two variables is explained within the framework of defence mechanisms which have to be integrated with environment factors such as information, the patient-family-health workers relationship and with the disease itself.

As an example, at the beginning of the chemotherapy some patients aware of colon cancer diagnosis denied the advanced phase with liver metastasis. This level of awareness together with the close relationship with the oncologist who supports and motivates the patient to chemotherapy, warded off anxiety and depression, promoting compliance to medical treatment.

### Anxiety and depression

were measured by the psychologist using the Hospital Anxiety and Depression (HAD) scale [30]. This self-rating scale is designed to detect states of anxiety and depression in patients with physical illnesses. HAD scores range from 0 to 21 for anxiety and for depression. Past studies have established that scores of greater than or equal to 8 for the depression scale or 10 for the anxiety scale are classified as a clinical case [31]. Scores from 0 to 7 indicate normal levels, 8 to 10 are regarded as borderline and 11 to 21 indicate severe anxiety or depression, i.e. psychiatric disorder [32].

### Subjective perception of medical treatment quality

The evaluation was made by the psychologist with a structured interview centred on patient perception of medical treatment.

Structured interviews were codified using a pre-designed questionnaire for data collection to record patient responses. No questionnaire on these variables was available. The content of the questionnaire was established using the results of a previous report on ACC patients [33]. Indeed, the ACC caring health workers were consulted to ensure that it included items considered relevant and valid to this expert group. The questionnaire consisted of both open and closed questions designed to elicit patient perception on treatment area (expectations regarding chemotherapy tumor response and side effects and modifications over time, preference of bolus versus infusional chemotherapy, perception of chemotherapy efficacy, length, interval between courses, impact on HRQOL) the patient area (perception of active participation, chemotherapy impact on anxiety, depression, interpersonal relationships, work, free-time) and the team area (perception of oncologist communication and information and of psychologist containment). Interviews were transcribed verbatim and each transcript reviewed for identification of common themes which described the experience of patients.

Interview variables were collected after 9 and 18 weeks of chemotherapy apart from expectations regarding response to treatment and toxicity which were also evaluated at baseline.

HRQOL: was assessed by the psychologist with the EORTC QLQ C30 questionnaire, using a validated Italian translation. This measure includes five functioning scales, one global health and HRQOL status scale, and eight symptom scales [34].

### Statistical analysis

Analyses to evaluate changes after 18 weeks in outcomes scores were performed using a non parametric test because of the small sample size and non-normal distribution of the data.

The McNemar test was used to investigate the difference of variables for anxiety and depression as measured with the HAD scale before and after 18 weeks of treatment. The same test was adopted to investigate the difference of structured interview variables between 9 and 18 weeks of therapy. The difference between the HRQOL, and HAD mean scores before and after 18 weeks of treatment was evaluated with paired-samples T-test and chi-square test. The probability level was p < 0.05. All statistical analyses were performed in SPSS.

## Results

During a five years period, 119 metastatic or locally ACC patients were enrolled in the study. Seven patients refused participation (3 were too sick and 4 were not interested in the psychological intervention), 2 were excluded because of brain metastases and 12 died before the first evaluation. The 4 patients who refused participation were encouraged to contact the medical oncologist or the psychologist for further psychological support. The analysis was therefore conducted on 98 patients. Patient data are shown in Table 1.

**Table 1:** Characteristics of the patients

Enrolled patients	119
Patients excluded for:	
patient refusal	7
brain metastasis	2
patient death	12
Valuable patients	98
Mean age (years)	58
(range)	(25 – 77)
Sex (M/F)	62/36
Performance Status (WHO)	
0–1	67
≥ 2	31
Primary tumor site:	
colorectal cancer	98
Education	
elementary school	24
lower school	24
upper school	28
degree	22
Marital status	
married	69
single	10
separated	9
widow/widower	10

Of 98 patients enrolled 95 were assessed for treatment response. 4 patients showed complete response (4 %), 34 partial response (36 %), 38 no change (40 %) and 19 progression (20 %). The psychopathologic disorders before treatment are reported in Table 2: 29 patients of the sample (98 pts) presented psychopathological disorders (30%).

**Table 2:** Psychopathologic disorders in 98 patients examined with the clinical interview.

**Psychopathologic disorders**	**Patients (%)**
Adjustment disorders	20 (21)
anxiety	4 (5)
depression	2 (1)
mixed emotional features	14 (15)
Phobias	3 (3)
Personality disorders	3 (3)
Generalized anxiety disorder	3 (3)
Major depression	0

**Figure 1:**
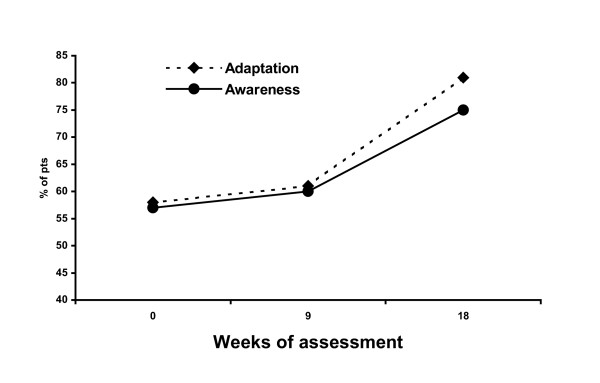
Modification of patient adaptation and awareness measured by the semistructured interview.

A total of 294 semi-structured psychodynamic interviews were conducted. A statistically significant improvement was observed in terms of adaptation and awareness between 0 and 18 weeks of therapy according to the McNemar test (p < 0.05), Figure 1.

Before initiating treatment the mean HAD score in the whole population was 4.9 ± 2.9 for anxiety and 5.5 ± 3.4 for depression, indicating the absence of abnormal anxiety and depression. However, there was a reduction in anxiety symptoms (p < 0.02) (Table 3) in the majority of subjects and the proportion of people with an anxiety score = 8 decreased from 19% to 10% (p < 0.02), Table 4. No significant difference was observed for depression before and after treatment.

**Table 3:** Changes in HAD scores after 18 weeks of therapy.

	**Pre-treatment score **mean ± SD	**Final score **mean ± SD	**p**
Anxiety	4.9 ± 2.9	4.3 ± 2.3	< 0.02
Depression	5.5 ± 3.4	5.2 ± 2.9	n.s

**Table 4:** Patients with normal or borderline/severe anxiety and depression measured by HAD scale before and after 18 weeks of treatment

	Normal patients	Borderline/Severe patients	p
**Anxiety**			
Pre-treatment	79	19	0.02
Post-treatment	88	10	
**Depression**			
Pre-treatment	85	23	n.s
Post-treatment	86	22	

After 18 weeks of chemotherapy the structured interview for the subjective perception of therapy and quality of care showed a significant increase in the percentage of patients who positively experienced the impact of treatment on HRQOL (53% Vs 70%), on anxiety (49% Vs 63%), on depression (54% Vs 69%), on interpersonal relationships (61% Vs 79%), on free-time (61% Vs 73%) and of those who had a positive perception of treatment quality (75% Vs 86%). As far as expectations regarding response and toxicity were concerned, after 18 weeks the proportion of people who experienced the treatment as efficient and without important side effects increased. A high percentage of patients positively experienced the team relationship modality during the whole course of treatment (92% Vs 93%), Table 5.

**Table 5:** Patients who positively experience the treatment by the structured interview

	**9 weeks *n***	**18 weeks *n***	***p***
**Treatment area**			
Impact on QoL	53	70	0.001
Expectations	68	75	n.s.
Modification over time	63	72	n.s.
Bolus vs. infusional	51	59	n.s.
Efficacy	93	95	n.s.
Side effects	45	54	n.s.
Length	47	42	n.s.
Interval between courses	78	78	n.s.
**Patient area**			
Impact on anxiety	49	63	0.03
Impact on depression	54	69	0.02
Interpersonal Relationship	60	78	0.003
Free-Time	60	72	0.03
Work	58	62	n.s.
Subjective perception of treatment quality	75	86	0.02
**Team area**			
Oncologist communication	90	90	n.s.
Psychologist containment	90	91	n.s.

From interview, the contents referred by the patients regarding relationship modality with the team was the need for information related to the status of disease, to treatment modality and future perspectives and the communication centred on sincere and reassuring relations. The interview results were utilised for planning future psychological interventions.

**Figure 2:**
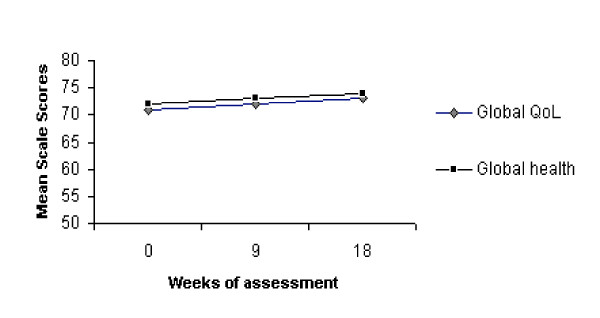
Evolution over time of the global health and QoL status measured by EORTC QLQ C30 questionnaire.

All the EORTC QLQ-C30 questionnaire scales mean scores were stable during the entire treatment (Figure 2). The lowest mean scores of the functioning scales were those of the emotive, Global Health and Global QoL status while the highest mean scores of the symptoms scales were those of fatigue.

## Discussion

The results of our study show that after four months of treatment, advanced colorectal cancer patients who were followed by an integrated team appeared more adapted and aware, less anxious, with a stable HRQOL, with a positive experience regarding medical therapy and satisfied with the integrated approach.

Possible explanations for an independent improvement of psychological variables include the integrated approach, the positive effect of palliative chemotherapy and the natural development of adaptation in a crisis situation [35]. Experimental and quasi-experimental studies demonstrate that individual or group psychological interventions improve emotional adjustment and interpersonal and social relationships, reduce emotional distress related to treatment and disease [36-38]. In a meta-analysis of 45 randomised trials, patients who received a psychological intervention had an improvement of 12% in emotional adjustment, 10% in psychosocial functioning and 14% in symptoms related to treatment and disease as opposed to the non intervention group [39]. Ferlic in particular, showed an improvement of adjustment, disease awareness and self-esteem in advanced cancer patients treated with an educational group counselling [40].

Our study sample has the same distribution of the adult Italian population in terms of social and demographic variables, including marital status. This latter (70% of our patients) is considered a positive prognostic factor [41]. All the patients enrolled in the present study had advanced colorectal cancer; 68% of-them had 0–1-performance status, according to WHO criteria and all received the same chemotherapy regimen.

The patient's psychological state before initiating treatment, showing a prevalence of normal anxiety and depression and a psychopathology in 30% of the cases, characterised principally by adaptation disorders (70% of the cases), is in agreement with Derogatis [42] and Massie [43].

According to Pinder, anxiety and depression in patients assessed with HADs, showed 19% of high anxiety scores [43]. During chemotherapy normal patients did not become distressed and pathological patients decreased. This seems to be the opposite of the significant increase of anxiety and depression in the non-intervention group of randomised clinical trials [44,45].

In the recent literature interest in patient care satisfaction is increasing. The major determinants of care satisfaction are: providing information, rapport and attention to patients needs [46,47]. The results obtained by the structured interviews on patient positive perception of treatment underline the adaptation to chronomodulated therapy after four months.

The positive inner experience of almost all of the patients with regard to the adequacy of the physician-patient relationship and the psychological containment suggest that the integrated approach is desired by the patients and feasible in routine clinical practice. Kiebert [48] and Hopwood [49] stressed the relevance of the physician-patient relationship based on clear explanations and emotional involvement. In haematological neoplasm, these variables influence the patient's subjective perception of the severity of the disease and acceptance [50]. It has always been known that interventions focusing on doctor-patient relationship improve the social support for the patient, and have a positive effect on HRQOL [48,49] and on adaptation to disease.

If our results could be confirmed in a randomised trial this integrated approach could become useful to foster active participation to treatment [51,52], to prevent the doctor-patient relationship crisis, to increase the compliance to therapy and to reduce the risk of psychopathological complications.

The EORTC QLQ C30 questionnaire confirmed the literature studies with regard to the effects of chemotherapy on advanced colon cancer patient HRQOL indicating that HRQOL remained stable during a four month period [53-58]. Before initiating treatment the deteriorate variables of HRQOL assessed with the EORTC QLQ questionnaire were emotive functioning, Global QoL status and fatigue. This could be the effect of the crisis related to the advanced disease phase and to the expectation of chemotherapy and an integrated approach may be important for an improvement in psychophysical well-being.

Although few conclusions can be drawn from the present study, the results do provide some evidence for the benefits of this psychological intervention for advanced colorectal cancer patients. This devastating situation often leads patients to psychological distress with a continual need for security, belonging and identity [59]. The psychological response to these needs must take into account the limited life expectancy of these patients (an average of 18–20 months) and must therefore focus on the best possible use of all the resources available and influence psychological distress in brief time periods. The importance of an intervention for all advanced patients treated in an out-patient service seems to be supported by studies where psychosocial variables are less important for the development of psychological disorders than the deteriorated health status due to the advanced phases of disease.

In the cancer setting, experience of primary care intervention is quite limited. Although we are aware of a high rate of psychopathological symptoms, only a limited number of cancer patients receive an adequate second level of assistance [60,61]. Thus, many cancer patients are not referred for psychological assistance at all, even if the severity of their symptoms is relevant [62,63,23]. In our opinion the necessity of primary care assistance for all cancer patients can not be procrastinated.

Our intervention employs resources already available in a hospital setting, i.e. the medical team. The "team" of this report is different from other medical teams, when we consider the inclusion of the psychologist from the first medical examination and for the entire duration of the treatment [34]. The health workers will be confronted with different object relationship modalities: this "model", based on ego psychology encompasses the nature of self – and object representations [64], the nature of object relations [65,66], this integrated approach which utilises a psychological understanding with a dynamic background regards both disciplines and provides the basis for bridging the gap between oncologist and psychologist.

The presence of the psychologist from the very beginning in an out-patient setting where the team takes care of the patient resulted in a good compliance with only seven patients refusing the psychological intervention, fewer than those reported in the literature [44,67,68]. Since this primary care assistance uses an integrated approach with a dynamic background involving a diversified psychological approach for each patient with advanced disease, it does not seem to share the biological, psychological and social limits of other educational and psychotherapeutic approaches [66].

The greatest limitation of our study is that it is not a randomised trial. It can not be demonstrated whether the psychological strategy we used was the key factor in improving patients' psychophysical well-being. Another limitation is the fact that a longitudinal follow-up study on advanced colon cancer patients to assess the long-term effects of the psychological treatment cannot be carried out due to the short survival rate of these patients. Further studies will be necessary to confirm these preliminary results.

## References

[B1] Loscalzo M, Brintzenhofeszoc K, Holland JC (1998). Brief crisis counseling. Psycho-oncology.

[B2] Ferlic M, Goldman A, Kennedy BJ (1979). Group counseling in adult patients with advanced cancer. Cancer.

[B3] Fawzy FI, Cousins N, Fawzy NW, Kemeny ME, Elashoff R, Morton D (1990). A structured psychiatric intervention for cancer patients: Changes over time in methods of coping and affective disturbance. Arch Gen Psychiatry.

[B4] Beck AT (1976). Cognitive therapy and the emotional disorders.

[B5] Moorey S, Greer S (1989). Psychological therapy for patients with cancer A new approach.

[B6] Worden JW, Weisman AD (1984). Preventive psychosocial intervention with newly diagnosed cancer patients. Gen Hosp Psychiatry.

[B7] Watson M, Fenlon D, McVey G, Fernandez Marcos M (1996). A support group for breast cancer patients: development of a cognitive-behavioural approach. Behav Cogn Psychother.

[B8] Bottomley A (1998). Psychotherapy groups and cancer patient survival: chasing fool's gold?. Eur J Cancer Care.

[B9] Alexander F, French TM (1946). Psychoanalytic therapy: Principles and applications.

[B10] Sifneos PE (1979). Short-term dynamic psychotherapy evaluation and technique.

[B11] Mann J (1973). Time-limited psychotherapy.

[B12] Malan D (1976). Toward the validation of dynamic psychotherapy: a replication.

[B13] Luborsky L (1984). Principles of psychoanalytic psychotherapy: A manual for supportive-expressive treatment.

[B14] Strupp HH, Binder J (1984). Psychotherapy in a new Key: A guide to time limited dynamic psychotherapy.

[B15] Crits-Christoph P, Barber JP (1991). Handbook of short-term dynamic psychotherapy.

[B16] Greer S, Moorey S, Baruch JD, Watson M, Robertson BM, Mason A, Rowden L, Law MG, Bliss JM (1992). Adjuvant psychological therapy for patients with cancer: a prospective randomised trial. BMJ.

[B17] Moorey S, Greer S, Watson M, Baruch JDR, Robertson BM, Mason A, Rowden L, Tunmore R, Law M, Bliss JM (1994). Adjuvant psychological therapy for patients with cancer: one year follow-up of a randomised controlled trial. Psychooncology.

[B18] Moorey S, Greer S, Bliss JM, Law M (1998). A comparison of adjuvant psychological therapy and supportive counselling in patients with cancer. Psychooncology.

[B19] Brown D, Pedder J (1991). Introduction to Psychotherapy. London: Routledge cancer Soc Sci Med.

[B20] Leshan L (1989). Cancer as a turning point.

[B21] Chiozza LA (1981). Psicoanalisi e cancro.

[B22] Roth A, Fonagy P (1996). Psicoterapie e prove di efficacia Quali terapie per quali pazienti.

[B23] Fallowfield L, Ratcliffe D, Jenkins V, Saul J (2001). Psychiatric morbidity and its recognition by doctors in patients with cancer. Br J Cancer.

[B24] Levi F (2001). Circadian chronotherapy for human cancers. Lancet Oncol.

[B25] Levi F, Zidani R, Misset JL (1997). Randomised multicentre trial of chronotherapy with oxiplatin, fluorouracil and folinic acid in metastatic colorectal cancer. Lancet.

[B26] Garufi C, Levi F, Aschelter AM, Pace R, Giunta S, Nistico C, Galla DA, Silecchia GF, Franchi F, Narduzzi C, Terzoli E (1997). A phase I trial of five-day chronomodulated infusion of 5-fluorouracil and 1-folinic acid in patients with metastatic colorectal cancer. Eur J Cancer.

[B27] Pavan L, Banon D (1996). Trauma, vulnerabilità, crisi.

[B28] American Psychiatric Association (1987). Diagnostic and Statistical Manual of Mental Disorders 3rd revised ed (DSM III-R).

[B29] Bottomley A (1998). Psychotherapy groups and cancer patient survival: chasing fool's gold?. Eur J Cancer Care.

[B30] Zigmond AS, Snaith RP (1983). The Hospital Anxiety and Depression scale. Acta Psychiat Scand.

[B31] Carroll BT, Kathol RG, Noyes R, Wald TG, Clamon GH (1993). Screening for depression and anxiety in cancer patients using the Hospital Anxiety and Depression Scale. Gen Hosp Psychiatry.

[B32] Watson M, Meyer L, Thomson A, Osofsky S (1998). Psychological factors predicting nausea and vomiting in breast cancer patients on chemotherapy. Eur J Cancer.

[B33] Pugliese P, Garufi C, Nisi E, Caruso A, Nistico C, Giunta S, Giannarelli D, Terzoli E (1995). Personality, Inner Experience and Compliance in Advanced Cancer Patients Treated with External Pumps. J Infus Chemother.

[B34] Aaronson NK, Ahmedzai S, Bergman B, Bullinger M, Cull A, Duez NJ, Filiberti A, Flechtner H, Fleishman SB, de Haes JC, Kaasa S, Klee M, Osoba D, Razavi D, Rofe PB, Schraub S, Sneeuw K, Sullivan M, Takeda F (1993). The European Organisation for the Research and Treatment of Cancer QLQ-C30: a HRQOL instrument for use in international clinical trials in oncology. J Natl Cancer Inst.

[B35] Lederberg M, Holland JC, Kaplan Hl, Sadok BJ (1995). Psycho-oncology. Comprehensive Textbook of Psychiatry.

[B36] Andersen BL (1992). Psychological interventions for cancer patients to enhance the HRQOL. J Consult Clin Psychol.

[B37] Bottomley A, Hunton S, Roberts G, Jones L, Bradley C (1996). A pilot study of cognitive behavioural therapy and social support group interventions with newly diagnosed cancer patients. J Psychosoc Oncol.

[B38] Trijsburg RW, van Knippenberg FC, Rijpma SE (1992). Effects of psychological treatment on cancer patients: a critical review. Psychosom Med.

[B39] Meyer TJ, Mark MM (1995). Effects of psychological interventions with adult cancer patients: a meta analysis of randomized experiments. Health Psychol.

[B40] Ferlic M, Goldman A, Kennedy BJ (1979). Group counselling in adult patients with advanced cancer. Cancer.

[B41] Goodwin JS, Hunt WC, Key CR, Samet JM (1987). The effect of marital status on stage, treatment and survival of cancer patients. JAMA.

[B42] Derogatis LR, Morrow GR, Fetting J, Penman D, Piasetsky S, Schmale AM, Henrichs M, Carnicke CL (1983). The prevalence of psychiatric disorders among cancer patients. JAMA.

[B43] Massie MJ, Holland JC, Holland JC, Rowland JH (1989). Overview of normal reactions and prevalence of psychiatric disorders. Handbook of Psychoncology.

[B44] Pinder KL, Ramirez AJ, Black ME, Richards MA, Gregory WM, Rubens RD (1993). Psychiatric disorders in patients with advanced breast cancer: prevalence and associated factors. Eur J Cancer.

[B45] Fawzy FI, Fawzy NW, Hyun CS, Elashoff R, Guthrie D, Fahey JL, Morton DL (1993). Malignant melanoma: effects of an early structured psychiatric intervention, coping and affective state on recurrence and survival six years later. Arch Gen Psychiatry.

[B46] Taenzer P, Bultz BD, Carlson LE, Speca M, DeGagne T, Olson K, Doll R, Rosberger Z. (2000). Impact of computerized quality of life screening on physician behaviour and patient satisfaction in lung cancer outpatients. Psychooncology.

[B47] Razavi D, Delvaux N (1997). Communication skills and psychological training in oncology. Eur J Cancer.

[B48] Kiebert GM, de Haes JC, van de Velde CJ (1991). The impact of breast-conserving treatment and mastectomy on the HRQOL of early-stage breast cancer patients: a review. J Clin Oncol.

[B49] Hopwood P, Thatcher N (1990). Preliminary experience with HRQOL evaluation in patients with lung cancer. Oncology.

[B50] Heinrich RL, Schag CC (1985). Stress and activity management: group treatment for cancer patients and their spouses. J Consult Clin Psychol.

[B51] Zittoun R (1982). L'information des malades en hematologie. Bordeaux Medicine.

[B52] Bloom JR (1982). Social support, accommodation to stress and adjustment to breast. Soc Sci Med.

[B53] Stefanek ME, Shaw A, DeGeorge D, Tsottles N (1989). Illness-related worry among cancer patients: Prevalence, severity and content. Cancer Invest.

[B54] Spiegel D (1994). Commentary. Psychosocial intervention in cancer. J Natl Cancer Inst.

[B55] Fallowfield LJ, Hall A, Maguire GP, Baum M (1990). Psychological outcomes of different treatment policies in women with early breast cancer outside a clinical trial. BMJ.

[B56] Scheithauer W, Rosen H, Kornek GV, Sebesta C, Depisch D (1993). Randomised comparison of combination chemotherapy plus supportive care with supportive care alone in patients with metastatic colorectal cancer. BMJ.

[B57] Glimelius B, Hoffman K, Graf W, Pahlman L, Sjoden PO (1994). Quality of life during chemotherapy in patients with symptomatic advanced colorectal cancer. The Nordic Gastrointestinal Tumor Adjuvant Therapy Group. Cancer.

[B58] Schoffski P, Kohne CH, Schellenberger U (1995). Does effective chemotherapy for metastatic colorectal cancer (CC) improve HRQOL (QL)? Preliminary results of a randomized phase II study using the EORTC QLQ C 30. Eur J Cancer.

[B59] Hill M, Norman A, Cunningham D, Findlay M, Watson M, Nicolson V, Webb A, Middleton G, Ahmed F, Hickish T (1995). Impact of protracted venous infusion fluorouracil with or without interferon alfa-2b in tumour response, survival and HRQOL in advanced colorectal cancer. J Clin Oncol.

[B60] Rougier P, Van Cutsem E, Bajetta E, Niederle N, Possinger K, Labianca R, Navarro M, Morant R, Bleiberg H, Wils J, Awad L, Herait P, Jacques C (1998). Randomised trial of irinotecan versus fluorouracil by continuous infusion after fluorouracil failure in patients with metastatic colorectal cancer. Lancet.

[B61] Cunningham D, Pyrhonen S, James RD, Punt CJ, Hickish TF, Heikkila R, Johannesen TB, Starkhammar H, Topham CA, Awad L, Jacques C, Herait P (1998). Randomised trial of irinotecan plus supportive care versus supportive care alone after fluorouracil failure for patients with metastatic colorectal cancer. Lancet.

[B62] Ford S, Fallowfield L, Lewis S (1994). Can oncologists detect distress in their out-patients and how satisfied are they with their performance during bad news consultations?. Br J Cancer.

[B63] Lampic C, Nordin K, Sjdén PO (1995). Agreement between cancer patients and their physicians in the assessment of patient anxiety at follow-up visits. Psycho-Oncology.

[B64] Hartmann H (1950). Comments on the psychoanalytic theory of the ego. Psychoanalytic study of the child.

[B65] Jacobson E (1954). The self and the object world: vicissitudes of their infantile cathexes and their influence on ideational and affective development. Psychoanalytic.

[B66] Kernberg O (1966). Structural derivatives of object relationships. Int J Psychoanal.

[B67] Ford MF, Jones M, Scannell T, Powell A, Coombes RC, Evans C (1990). Is group psychotherapy feasible for oncology out-patient attenders selected on the basis of psychological morbidity?. Br J Cancer.

[B68] Baider L, Amikam JC, De-Nour AK (1984). Time-limited thematic group with post-mastectomy patients. J Psychosom Res.

